# Effects of high-intensity interval training on improving arterial stiffness in Chinese female university students with normal weight obese: a pilot randomized controlled trial

**DOI:** 10.1186/s12967-022-03250-9

**Published:** 2022-02-02

**Authors:** Jingyun Hu, Min Liu, Ruoyu Yang, Liyan Wang, Leichao Liang, Yuanyuan Yang, Shihao Jia, Ruiyi Chen, Qianle Liu, Yu Ren, Lei Zhu, Ming Cai

**Affiliations:** 1 Central Lab, Shanghai Pudong New Area People’s Hospital, 490 Chuanhuan South Road, Shanghai, 201299 China; 2 College of Sport Sciences, Qufu Normal University, Qufu, 273165 Shandong Province China; 3 College of Rehabilitation Science, Shanghai University of Medicine & Health Sciences, 257 Tianxiong Road, Shanghai, 201318 China; 4 Shanghai University of Medicine & Health Sciences Affiliated Zhoupu Hospital, 1500 Zhouyuan Road, Shanghai, 201318 China

**Keywords:** High-intensity interval training, Normal weight obese, Female university students, Arterial stiffness, Body composition

## Abstract

**Background:**

High intensity interval training (HIIT) has been reported to exert better effects on cardiovascular fitness in obesity, but little known about the arterial stiffness (AS) in female university students with normal weight obesity (NWO). Thus, this study aimed to investigate the effects of HIIT on the body composition, heart rate (HR), blood pressure (BP), blood lipids metabolism as well as the novel parameters of propensity for AS (arterial velocity pulse index [AVI], arterial pressure volume index [API]) for female university students with NWO.

**Methods:**

Forty female university students with NWO were randomly assigned to control group (*n* = 20) and HIIT group (3 bouts of 9‑min intervals at 90% of the maximal heart rate [HR_max_], interspersed by 1 min rest, 5 days a week, *n* = 20). Tests were performed before and after 4 weeks of training. Repeated measures ANOVA and simple effect test analysis were used to analyze dependent variable changes.

**Results:**

After 4 weeks HIIT statistically significantly improved the body composition by decreasing the body mass index, body fat percent, total body fat mass (BFM), BFM of left arm, measured circumference of left arm, and obesity degree, and increasing the total body skeletal muscle mass, protein content, total body water, fat free mass, body cell mas, and InBody score. HIIT also statistically significantly decreased the HR and BP. As for the lipid profile, HIIT obviously ameliorated the blood lipids metabolism by decreasing the levels of total cholesterol (TC), triglyceride, low-density lipoprotein, and TC/HDL, and increasing the levels of high-density lipoprotein (HDL). In addition, the AVI and API were markedly decreased via HIIT intervention.

**Conclusions:**

HIIT produced significant and meaningful benefits for body composition, HR, BP, and blood lipids metabolism, and could decrease AS in female university students with NWO. This suggests that HIIT may effectively reduce the risk of arteriosclerosis and protect the cardiovascular function for female university students with NWO.

*Trial registration* ChiCTR2100050711. Registered 3 September 2021. Retrospectively registered.

## Background

Obesity, as a disease and an independent risk factor for cardiovascular disease (CVD), has an annually increased global incidence. Traditionally, body mass index (BMI) is the most popular indicator used in obesity classification, but it fails to consider and distinguish body composition variables such as body fat percentage (BF%), lean mass, and body fat distribution. Therefore, a BMI indicator is prone to cause imprecise judgment and underestimate obesity prevalence, especially for individuals with “normal weight obesity (NWO)” whose body weight and BMI (18.5–24.9 kg/m^2^) are in normal ranges but whose BF % is excessive (> 30%) [1]. It has previously been shown that the incidence of Chinese NWO was high among university students, especially in females, which might be related to estrogen [2]. The main barriers for university students to obtain a healthy lifestyle were a busy schedule, academic stress, a lack of time to exercise resulting in reduced physical activity/hypokinesia/sedentary behavior, overeating/hypercaloric diets/irregular diet patterns, and a lack of sleep/irregular sleeping patterns [3–5]. Additional studies have reported that Chinese university students with NWO have lower skeletal muscle mass and levels of physical fitness and higher cardiometabolic risk, especially in females [6, 7]. Thus, female university students with NWO should be given enough attention to enhance their physical and mental health.

NWO is mainly defined using BF%, which indicates an increased amount of fat in the abdominal region, known as visceral adipose tissue (VAT). VAT is a major determinant of the occurrence of metabolic disorders and could increase the risk of developing CVD compared with general obesity [8, 9]. Hence, identifying individuals with NWO as early as possible is an effective component for obesity prevention and treatment and will dramatically lower the occurrence of atherosclerosis and CVD in the future. Recent studies have suggested that individuals with NWO often show symptoms of cardiovascular metabolic disorders and have higher rates of atherosclerosis than subjects with normal BMI and normal amounts of body fat. Evidence described above implies that individuals with NWO are more likely to experience future cardiovascular events [1, 10]. Arterial stiffness (AS) is one of the first indicators detected in both functional and structural changes of the arterial wall and plays an important role in CVD; AS is also considered to be an independent risk predictor of atherosclerosis and CVD [11]. Unfortunately, AS has a very close association with NWO, and it has a strongly positive correlation with numerous NWO-induced anomalous parameters, such as higher levels of systolic blood pressure, VAT, triglyceride (TG), and low-density lipoprotein-cholesterol (LDL), in contrast to its negative correlation with a lower level of high-density lipoprotein-cholesterol (HDL) [12].

Currently, noninvasive devices and methods such as the cardio-ankle vascular index (CAVI), brachial-ankle pulse wave velocity (baPWV), and carotid artery intima-media thickness (IMT) are used to evaluate AS and atherosclerosis in hypertensive patients. However, these approaches require a relatively long period of time to complete measurements, and problems can be encountered in the skill and experience levels of the persons conducting the measurements. Moreover, these instruments are uncomfortable for patients because occlusion/sensing cuffs attached to both the arms and ankles must be used in the supine position [13]. Simpler and easier methods that can be used in daily clinical settings are required. In recent years, the arterial pressure volume index (API) and arterial velocity pulse index (AVI), which can be determined in a simple and noninvasive manner, have been used as new novel indices for AS. The AVI is an index based on the ratio between velocity changes during brachial artery dilatation (systolic phase) and relaxation (diastolic phase), which reflects aortic elasticity and peripheral vascular resistance. The principle of the API is that soft arteries with greater elasticity show change in volume more rapidly when the cuff pressure is reduced, which reflects the elasticity of the brachial artery and is considered to indicate the stage of atherosclerosis of the peripheral arteries [14, 15]. In a healthy adult population, AVI and API values were significantly elevated in individuals with classical cardiometabolic and atherosclerotic risk factors (e.g., obesity, hypertension, diabetes, and dyslipidemia) and showed a positive correlation with IMT or the CAVI, positive correlations with as well as baPWV and preclinical carotid atherosclerosis regardless of the presence or absence of coronary artery disease, drawing attention to the usefulness of the AVI and API [15–17]. Compared with conventional indices, they require shorter measurement times (approximately 1 min), have more comfortable postural requirements (in a sitting position), and involve easier operation of the measurement devices. These merits make the AVI and API fit for use in screening preclinical atherosclerosis in broad clinical and public health settings. However, to date, studies on the AVI and API have been performed mainly in Japan, and there have been no reports about using them to evaluate the AS of female university students with NWO in China.

Although increasing evidence suggests that exercise is the most predominant nonpharmacological strategy for attenuating excessive VAT deposition and fighting obesity and related complications [18], female university students with NWO prefer diet to exercise. Among the main barriers for inactive individuals to physical activity and maintaining a physically active lifestyle are the reported lack of time, access to sporting facilities, the financial costs of exercising, and the inability to adhere to lengthy exercise programs. Recently, as an emerging and promising, highly efficient, multistimulated, cost-saving means and time-effective pattern of exercise intervention, high-intensity interval training (HIIT) has come at the right moment. HIIT involves several short sets of high-intensity bursts (≥ 80% of HRmax or ≥ 90% of maximal oxygen uptake, often reaching the anaerobic threshold) interspersed with low-intensity recovery or rest (65–80% of HRmax) [19, 20]. A growing body of evidence has demonstrated that HIIT can be an effective alternative to traditional moderate-intensity continuous training (MICT). Compared with MICT, HIIT has unique advantages in treating overweight/obese people because it not only can lead to greater adipose tissue loss, more effectively reduce abdominal and visceral fat mass, and improve body composition and dyslipidemia [21–23], but can also mediate larger decreases in systolic blood pressure [24], improve cardiorespiratory and vascular function, and reduce risk factors for CVD [21, 25]. Moreover, it is convenient and enjoyable for the public since the sport events and modes of HIIT vary and are not limited to a specific stadium [26, 27]. As a result, HIIT has high unsupervised adherence rates in overweight and obese adults. However, despite clear evidence for the positive vascular function and cardiovascular adaptations following HIIT, it is still unclear whether HIIT yield a beneficial effect on the AVI/API and AS by improving the body composition and lipid metabolism of female university students with NWO.

In this study, we selected female university students with NWO as the research object, mainly aiming to evaluate the possible changes in their body composition and cardiovascular function, and then investigated and analyzed the potential clinical effectiveness of short-term HIIT in the treatment of the changes described above.

## Materials and methods

### Study participants

We enrolled one hundred and thirty-seven female university students with normal BMI (18.5–24.9 kg/m^2^) at the Shanghai University of Medicine and Health Sciences (Shanghai, China) from November to December 2020. The experiment protocol was approved by the institutional review board of the Shanghai University of Medicine and Health Sciences (No. 2020-20YJCZH001-03), and all participants signed an informed consent form after understanding the experimental requirements and procedures, and they could withdraw from the study at any stage. The study was performed in accordance with the principles outlined in the Declaration of Helsinki.

Exclusion criteria were as follows: (1) physical limitations (e.g., musculoskeletal system injuries, osteoarticular diseases or pain); (2) known cardiovascular or cerebrovascular diseases; (3) exercise-related dyspnea; (4) known family history of sudden death, medication history or is taking any medications, uncontrolled hypertension, smoking, alcohol consumption, and type I or II diabetes; (5) BMI < 18.5 kg/m^2^ or > 24.9 kg/m^2^; (6) who reported engaging in competitive exercise or take part in a structured exercise program.

**Fig. 1:**
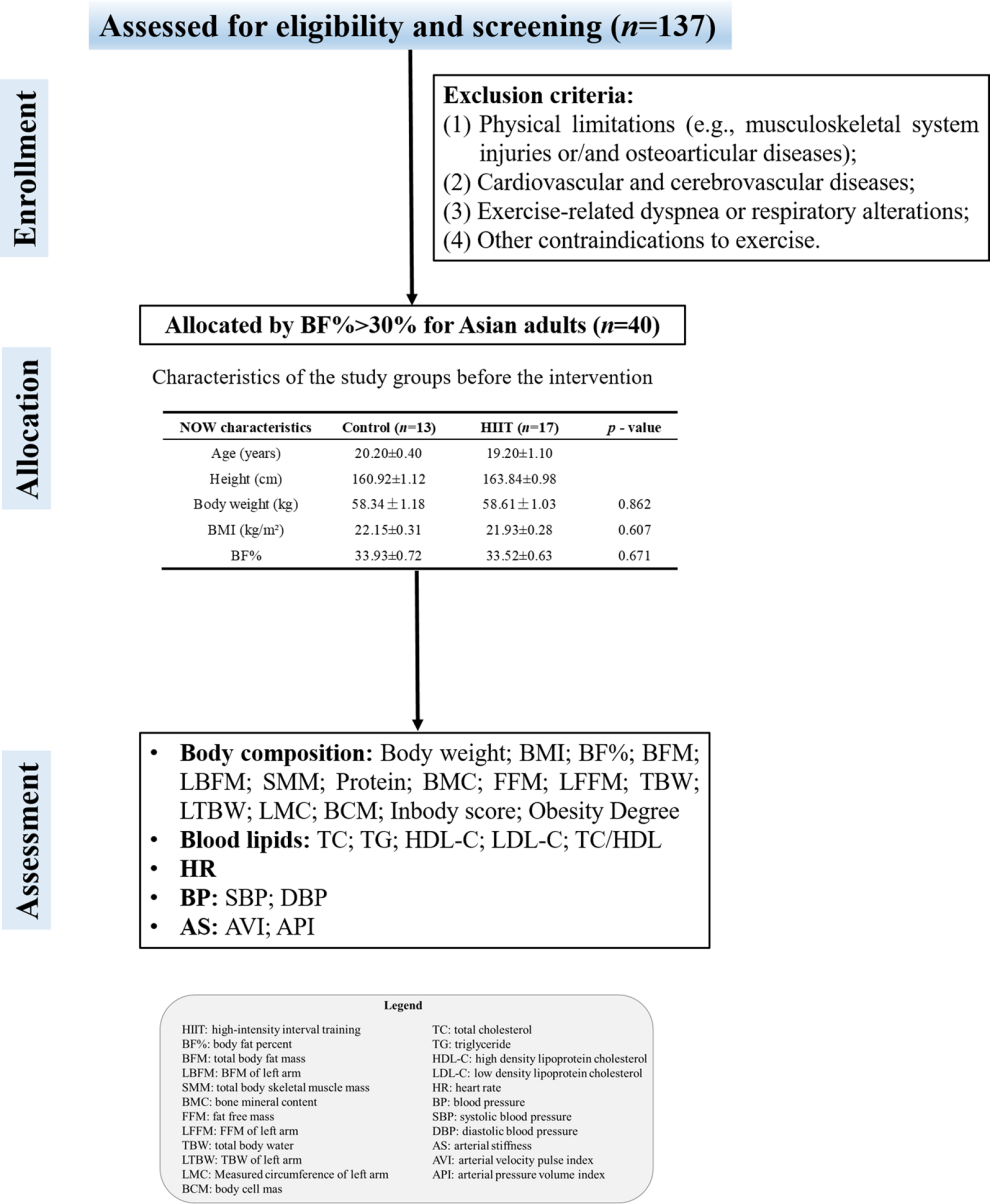
Flow chart diagram. The recruiting and screening process of normal weight obesity (NWO) female university students for study protocol. The assessment indicators include the body composition, lipid profile, and cardiovascular function

After the exercise risk assessment, forty NWO subjects were screened using body composition measurement apparatus InBody-770 (Biospace Co., Seoul, Korea) according to the criteria of female NWO (BF% > 30%, which is the NWO obesity cut-off for Asian adults) [1]. Participants were randomly divided into Control group (n = 20, age 20.20 ± 0.40) and HIIT group (n = 20, age 19.20 ± 1.10). The Control group only took participated in one health education each week, while the HIIT group performed duration of 4-week HIIT intervention. Both groups were instructed to maintain their habitual dietary patterns (and not to use any dietary supplements) and take two mandated physical education classes per week throughout the study. 10 of the 40 participants (7 in the control group and 3 in the HIIT group) resigned from the study during the intervention due to personal reasons. Flow-chart diagram is detailed in Fig. 1.

### HIIT Intervention protocol

**Fig. 2:**
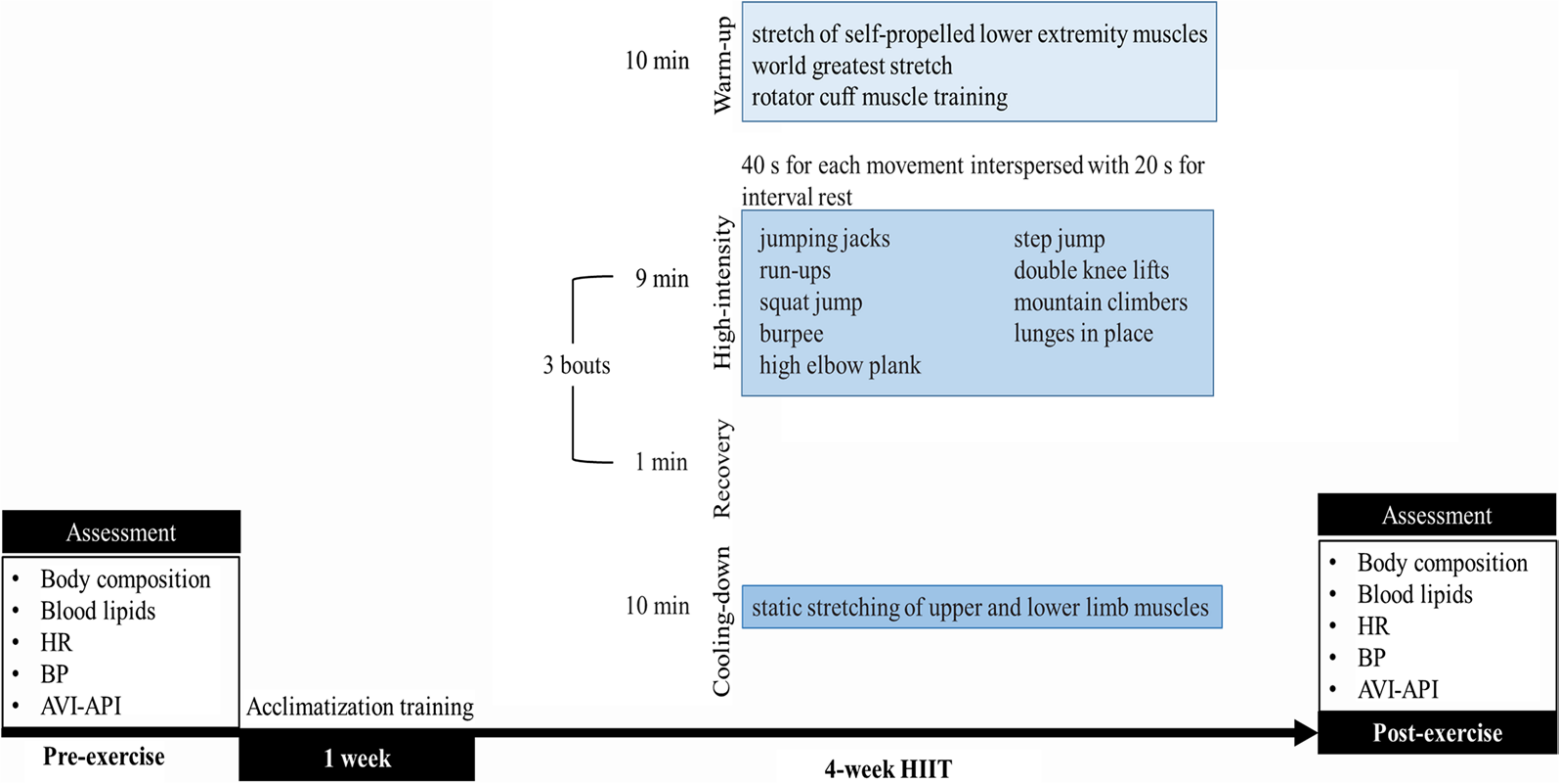
HIIT intervention protocol. HIIT was performed with 9 min of high-intensity exercise combined motion (40 s for each movement interspersed with 20 s for interval rest) followed by 1 min of recovery rest and this was repeated 3 bouts and ended with 10 min static stretching, five times a week. After 4-week, the body composition, lipid profile, and cardiovascular function were detected to observe the effects of HIIT

Participants in the HIIT group were familiarized with a week acclimatization training to acquaint the training session, learn the movements, and gradually adapt and increase the training intensity. After familiarization, the participants completed 4 weeks of training, and the frequency was five times per week. The HIIT intervention began with 10 min of warm-up training (muscle active stretching and joint mobility) to prevent exercise-related injuries. Then the training was performed with 9 min of high-intensity exercise combined motion (jumping jacks, run-ups, squat jump, burpee, high elbow plank, step jump, double knee lifts, mountain climbers, lunges in place; 40 s for each movement interspersed with 20 s for interval rest) followed by 1 min of recovery rest and this was repeated 3 bouts and ended with 10 min static stretching of upper and lower limb muscles. The intensity of training corresponded to 90% of the maximum heart rate (HR_max_) which was monitored by Prince-100H (Heal Force, Shanghai, China) during the entire training session. The entire HIIT process is guided by professional coaches and supervised by athletic instructor in case of exercise-related injuries or exercise fatigue. HIIT intervention protocol is detailed in Fig. 2.

## Measurements

### Body composition

Subjects were given complete instructions on the body composition analysis procedure and instructed not to make any intense physical activity for 8 h prior to body composition measurement. Body composition (body weight; BMI; BF%, body fat percent; BFM, total body fat mass; LBFM, BFM of left arm; SMM, total body skeletal muscle mass; protein; BMC, bone mineral content; FFM, fat free mass; LFFM, FFM of left arm; TBW, total body water; LTBW, TBW of left arm; LMC, measured circumference of left arm; BCM, body cell mas; InBody score, and obesity degree) was detected by InBody-770 multi-frequency bioelectrical impedance (BIA) device before and after the 4-week HIIT intervention. Before BIA measurement, the subjects were asked to take off their shoes, socks, hats, and heavy coats. Electronic products (e.g., mobile phones, earphones) and finger ring were also not permitted to carry. The subjects’ palms and the soles of their feet were wiped with an electrolyte tissue, then the subjects grasped the handle, placed their fingers in the standard locations, and abducted the upper limb 30° during the measurement.

### Blood lipids

All patients fasted for 8 h prior to examination. Fingertip blood samples (35 μL for each subject) were collected by capillary tube. Then they were added to a blood lipids test card quickly and examined by the three-in-one Cholesterol Monitoring system CCM-111 (Acon Biotech, Hangzhou, China) to detect the concentration of lipid profiles (TC, total cholesterol; TG; HDL; LDL; and TC/HDL) using the dry-chemistry method.

### Resting heart rate, blood pressure, and arterial stiffness

The left arm of resting heart rate (HR), systolic blood pressure (SBP), diastolic blood pressure (DBP), AVI, and API were all simultaneously measured using the PASESA AVE-2000 PLUS (Shisei Datum, Tokyo, Japan) with the subject in the sitting position. Measurements were taken in a quiet room with the temperature maintained at 20–26 ℃. The above parameters were calculated before the intervention and the 7:00 am the day after HIIT completion. The subjects were forbidden drinking coffee and kept calm and stable during the measurement for avoiding the fluctuation of blood pressure.

### Statistical analysis

All data are expressed as mean ± standard error (SE). The repeated measures ANOVA (group main effect and time by group interaction) within-between interaction design was used to evaluate indexes changes pre to post intervention through SPSS 26.0 software. If the interaction between time and group is significant, simple effect test analysis is needed. 95% confidence intervals (CIs) were examined for within-group changes. Statistical significance was set at p < 0.05.

## Results

The baseline characteristics of the participants were presented in Table 1. No differences were found between groups for any variable. Additionally, no adverse events occurred during the intervention period.

**Table 1:** Descriptive participant characteristics

	Control (*n* = 13)	HIIT (*n* = 17)	*p-*value
Age (years)	20.20 ± 0.40	19.20 ± 1.10	
Height (cm)	160.92 ± 1.12	163.84 ± 0.98	
Body weight (kg)	58.34 ± 1.18	58.61 ± 1.03	0.862
BMI (kg/m^2^)	22.15 ± 0.31	21.93 ± 0.28	0.607
BF%	33.93 ± 0.72	33.52 ± 0.63	0.671

Data are presented as mean ± standard error (SE)

*BMI* body mass index, *BF%* body fat percentage, *HIIT* high-intensity interval training

### Effects of HIIT on body composition

As shown in Table 2, we found a significant time × group interaction in the aspects of body weight (F_(1,28)_ = 21.348, p < 0.001, η^2^ = 0.433), BMI (F_(1,28)_ = 11.249, p = 0.002, η^2^ = 0.287), BF% (F_(1,28)_ = 46.558, p < 0.001, η^2^ = 0.624), BFM (F_(1,28)_ = 68.409, p < 0.001, η^2^ = 0.710), LBFM (F_(1,28)_ = 94.801, p < 0.001, η^2^ = 0.772), SMM (F_(1,28)_ = 9.599, p = 0.004, η^2^ = 0.255), protein content (F_(1,28)_ = 6.298, p = 0.018, η^2^ = 0.184), BMC (F_(1,28)_ = 4.928, p = 0.035, η^2^ = 0.150), LMC (F_(1,28)_ = 5.199, p = 0.030, η^2^ = 0.157), TBW (F_(1,28)_ = 9.230, p = 0.005, η^2^ = 0.248), FFM (F_(1,28)_ = 8.984, p = 0.006, η^2^ = 0.243), BCM (F_(1,28)_ = 9.453, p = 0.005, η^2^ = 0.252), obesity degree (F_(1,28)_ = 10.698, p = 0.003, η^2^ = 0.276), and InBody score (F_(1,28)_ = 26.614, p < 0.001, η^2^ = 0.487). However, there was no significant interaction effect in the LTBW (F_(1,28)_ = 2.822, p = 0.104, η^2^ = 0.092) and LFFM (F_(1,28)_ = 3.283, p = 0.081, η^2^ = 0.105). No significant difference was seen in the above indexes between the two groups before HIIT intervention. Simple effect test analysis found that compared with the Control group after HIIT intervention, BMI (F_(1,28)_ = 12.560, p = 0.001, η^2^ = 0.310), BF% (F_(1,28)_ = 92.648, p < 0.001, η^2^ = 0.768), BFM (F_(1,28)_ = 42.303, p < 0.001, η^2^ = 0.602), LBFM (F_(1,28)_ = 47.207, p < 0.001, η^2^ = 0.628), LMC (F_(1,28)_ = 6.641, p = 0.016, η^2^ = 0.192), and obesity degree (F_(1,28)_ = 12.476, p = 0.001, η^2^ = 0.308) were markedly decreased, SMM (F_(1,28)_ = 6.061, p = 0.020, η^2^ = 0.178), protein content (F_(1,28)_ = 4.900, p = 0.035, η^2^ = 0.149), TBW (F_(1,28)_ = 6.438, p = 0.017, η^2^ = 0.187), FFM (F_(1,28)_ = 6.214, p = 0.019, η^2^ = 0.182), BCM (F_(1,28)_ = 6.091, p = 0.020, η^2^ = 0.179), and InBody score (F_(1,28)_ = 42.337, p < 0.001, η^2^ = 0.602) were significantly increased in the HIIT group. There were no differences in body weight (F_(1,28)_ = 1.939, p = 0.175, η^2^ = 0.065), BMC (F_(1,28)_ = 3.966, p = 0.056, η^2^ = 0.124), LTBW (F_(1,28)_ = 0.753, p = 0.393, η^2^ = 0.026), and LFFM (F_(1,28)_ = 0.809, p = 0.376, η^2^ = 0.028) between the two groups.

**Table 2:** Effects of HIIT on body composition

	Control (*n* = 13)		HIIT (*n* = 17)		F		p—value		η^2^	
	Pre	Post	Pre	Post	Pre	Post	Pre	Post	Pre	Post
Body weight (kg)	58.34 ± 1.18	59.85 ± 1.24^△△^	58.61 ± 1.03	57.56 ± 1.08^##^	0.031	1.939	0.862	0.175	0.001	0.065
BMI (kg/m^2^)	22.15 ± 0.31	23.21 ± 0.46^△^	21.93 ± 0.28	21.03 ± 0.41^**#^	0.270	12.560	0.607	0.001	0.010	0.310
BF (%)	33.93 ± 0.72	35.55 ± 0.56^△^	33.52 ± 0.63	28.34 ± 0.49^**##^	0.184	92.648	0.671	0.000	0.007	0.768
BFM (kg)	19.79 ± 0.58	21.34 ± 0.58^△△^	19.67 ± 0.51	16.32 ± 0.51^**##^	0.027	42.303	0.870	0.000	0.001	0.602
LBFM (kg)	1.41 ± 0.05	1.56 ± 0.05^△△^	1.38 ± 0.04	1.10 ± 0.04^**##^	0.239	47.207	0.628	0.000	0.008	0.628
SMM (kg)	20.69 ± 0.53	20.64 ± 0.49	20.87 ± 0.46	22.25 ± 0.43^**##^	0.065	6.061	0.800	0.020	0.002	0.178
protein (kg)	7.51 ± 0.17	7.55 ± 0.17	7.61 ± 0.15	8.04 ± 0.15^*##^	0.180	4.900	0.675	0.035	0.006	0.149
BMC (kg)	2.39 ± 0.06	2.37 ± 0.05	2.39 ± 0.05	2.51 ± 0.05^##^	0.001	3.966	0.978	0.056	0.000	0.124
FFM (kg)	38.55 ± 0.88	38.51 ± 0.83	38.95 ± 0.77	41.24 ± 0.72^*##^	0.116	6.214	0.735	0.019	0.004	0.182
LFFM (kg)	1.71 ± 0.05	1.73 ± 0.06	1.69 ± 0.04	1.80 ± 0.05^##^	0.106	0.809	0.747	0.376	0.004	0.028
TBW (kg)	28.18 ± 0.65	28.18 ± 0.60	28.51 ± 0.57	30.21 ± 0.53^*##^	0.147	6.438	0.704	0.017	0.005	0.187
LTBW (kg)	1.33 ± 0.04	1.35 ± 0.05	1.31 ± 0.04	1.40 ± 0.04^##^	0.057	0.753	0.814	0.393	0.002	0.026
LMC (cm)	27.98 ± 0.26	28.52 ± 0.41	27.68 ± 0.22	27.12 ± 0.36^*^	0.785	6.641	0.383	0.016	0.027	0.192
BCM (kg)	24.92 ± 0.57	24.87 ± 0.54	25.13 ± 0.50	26.64 ± 0.47^*##^	0.079	6.091	0.781	0.020	0.003	0.179
Inbody score	68.85 ± 0.74	68.08 ± 0.69	68.94 ± 0.65	74.00 ± 0.60^**##^	0.009	42.337	0.924	0.000	0.000	0.602
Obesity degree	105.62 ± 1.53	110.46 ± 2.21^△^	104.47 ± 1.34	100.12 ± 1.93^**#^	0.318	12.476	0.578	0.001	0.011	0.308

Repeated measures ANOVA followed by simple effect test analysis were used

Data were presented as mean ± SE

*BMI* Body mass index, *BF* Body fat, *BFM* Body fat mass, *LBFM* BFM of left arm, *SMM* Skeletal muscle mass, *BMC* Bone mineral content, *FFM* Fat free mass, *LFFM* FFM of left arm, *TBW* Total body water, *LTBW* Total body water of left arm, *LMC* Measured circumference of left arm, *BCM* Body cell mas

^*^p < 0.05, **p < 0.01, HIIT group (post) vs. Control group (post)

^#^p < 0.05, ^##^p < 0.01, pre vs. post in HIIT group

^△^p < 0.05, ^△△^p < 0.01, pre vs. post in Control group

Pre- and post-intervention, the within-group comparisons showed that HIIT group had significantly decreased body weight (p = 0.007), BMI (p < 0.001), BF% (p < 0.001), BFM (p < 0.001), LBFM (p < 0.001), and obesity degree (p = 0.030), but had significantly increased SMM (p < 0.001), protein content (p < 0.001), BMC (p = 0.008), TBW(p < 0.001), LTBW(p = 0.002), FFM (p < 0.001), FFM of left arm (p = 0.002), BCM (p < 0.001), and InBody score (p < 0.001), no significant difference in LMC. Meanwhile, Control group had markedly increased body weight (p = 0.001), BMI (p = 0.040), BF% (p = 0.040), BFM (p = 0.002), LBFM (p < 0.001), and obesity degree (p = 0.030), no significant difference in SMM, protein content, BMC, TBW, LTBW, FFM, FFM of left arm, LMC, BCM, and InBody score.

### Effects of HIIT on heart rate and blood pressure

**Fig. 3:**
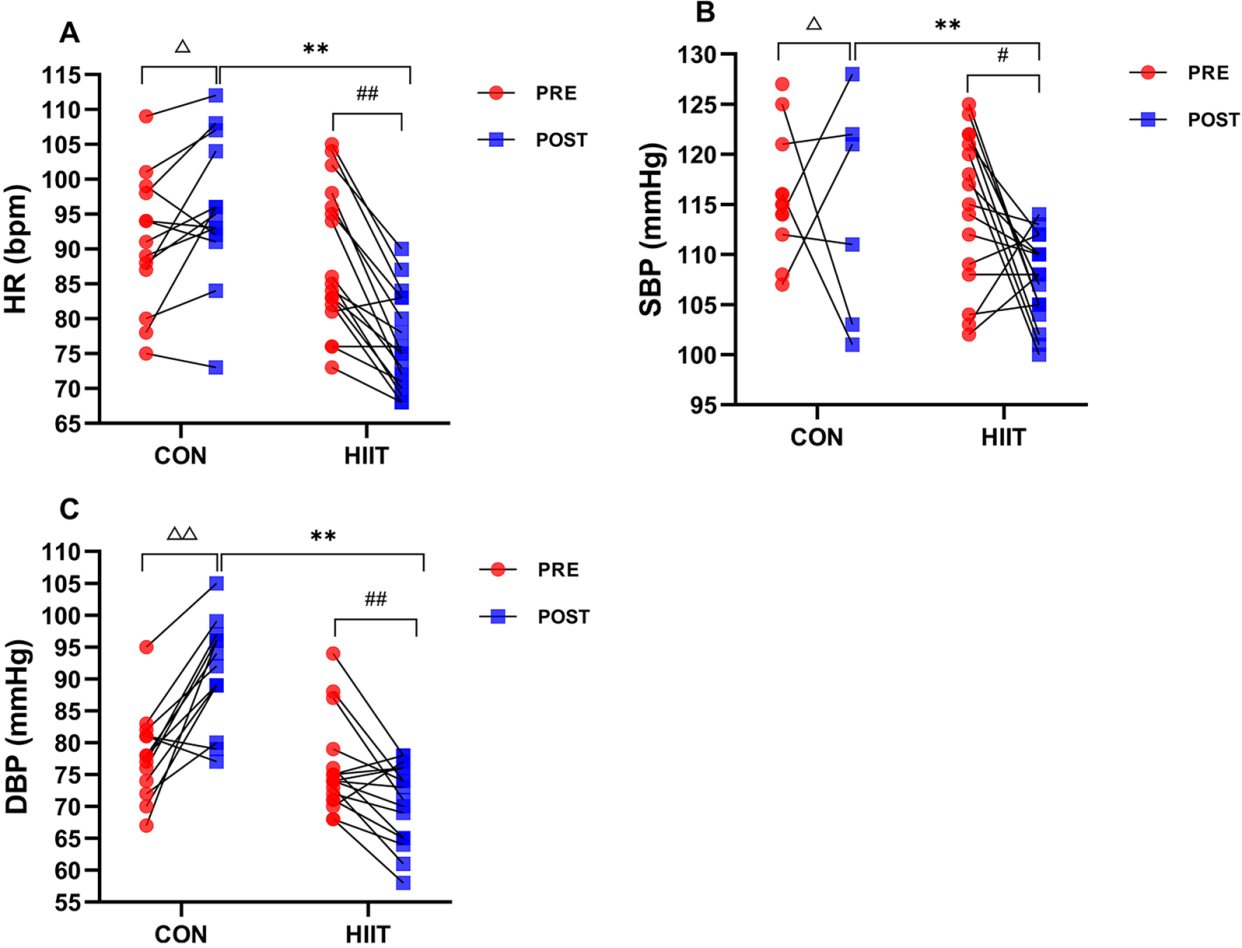
Effects of HIIT on heart rate and blood pressure. Changes of **A** HR (heart rate), **B** SBP (systolic blood pressure), and **C** DBP (diastolic blood pressure) were examined pre- and post-experiment using PASESA AVE-2000 PLUS. n = 13 Control group, n = 17 HIIT group. Repeated measures ANOVA followed by simple effect test analysis were used. Data are presented as mean ± SE. ^*^p < 0.05, ^**^p < 0.01, HIIT group (post) vs. Control group (post); ^#^p < 0.05, ^##^p < 0.01, pre vs. post in HIIT group; ^△^p < 0.05, ^△△^p < 0.01, pre vs. post in Control group

We found a significant time × group interaction in the aspects of resting HR (F_(1,28)_ = 37.780, p < 0.001, η^2^ = 0.574), SBP (F_(1,28)_ = 23.125, p < 0.001, η^2^ = 0.452), and DBP (F_(1,28)_ = 41.165, p < 0.001, η^2^ = 0.595). There was no significant difference in HR, SBP, and DBP between the two groups pre-HIIT intervention. However, simple effect test analysis found that compared with the control subjects post-HIIT intervention, the HR (F_(1,28)_ = 36.682, p < 0.001, η^2^ = 0.564, Fig. 3A), SBP (F_(1,28)_ = 24.624, p < 0.001, η^2^ = 0.468, Fig. 3B), and DBP (F_(1,28)_ = 60.632, p < 0.001, η^2^ = 0.684, Fig. 3C) were obviously decreased in HIIT group. Pre- and post-intervention, the within-group comparisons showed that HIIT group had significantly decreased HR (p < 0.001), SBP (p = 0.029), and DBP (p = 0.006), while the Control group had exactly opposite significant changes (Fig. 3A–C).

### Effects of HIIT on blood lipids

**Fig. 4:**
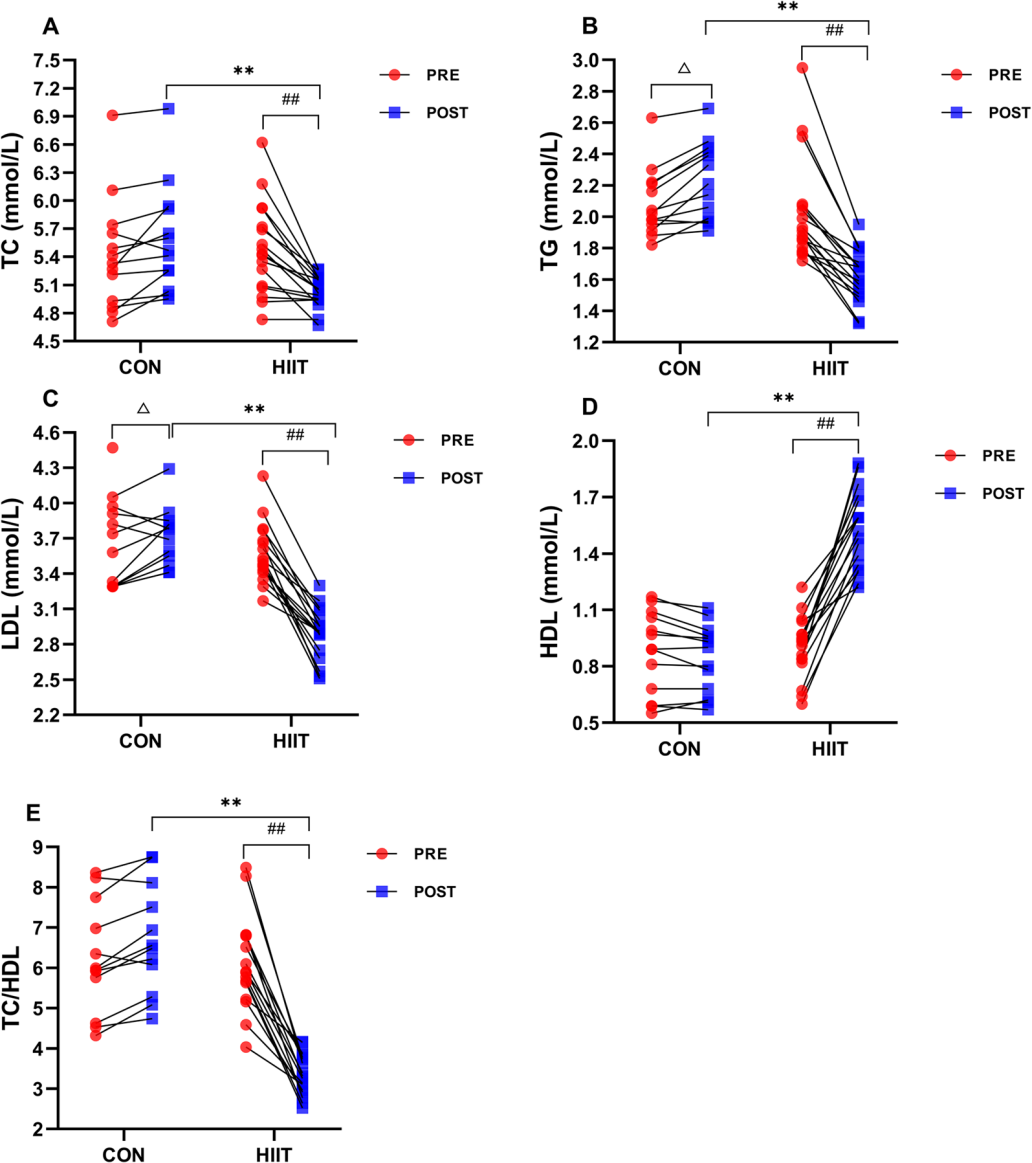
Effects of HIIT on blood lipids. Changes of **A** TC (total cholesterol), **B** TG (Triglyceride), **C** LDL (Low-density lipoprotein), **D** HDL (High-density lipoprotein), and **E** TC/HDL (Total cholesterol/High-density lipoprotein) were examined pre- and post-experiment using Cholesterol Monitoring system CCM-111. n = 13 Control group, n = 17 HIIT group. Repeated measures ANOVA followed by simple effect test analysis were used. Data are presented as mean ± SE. ^*^p < 0.05, ^**^p < 0.01, HIIT group (post) vs. Control group (post); ^##^p < 0.01, pre vs. post in HIIT group; ^△^p < 0.05, pre vs. post in Control group

We found a significant time × group interaction in the aspects of TC (F_(1,28)_ = 28.282, p < 0.001, η^2^ = 0.503), TG (F_(1,28)_ = 59.777, p < 0.001, η^2^ = 0.681), HDL (F_(1,28)_ = 103.813, p < 0.001, η^2^ = 0.788), LDL (F_(1,28)_ = 96.371, p < 0.001, η^2^ = 0.775), and TC/HDL (F_(1,28)_ = 81.708, p < 0.001, η^2^ = 0.745). There was no significant difference in HR, SBP, and DBP between the two groups before the HIIT intervention. However, simple effect test analysis found that compared with the control subjects post-HIIT intervention, the levels of TC (F_(1,28)_ = 14.882, p = 0.001, η^2^ = 0.347, Fig. 4A), TG (F_(1,28)_ = 65.006, p < 0.001, η^2^ = 0.699, Fig. 4B), LDL (F_(1,28)_ = 73.401, p < 0.001, η^2^ = 0.724, Fig. 4C), and TC/HDL (F_(1,28)_ = 86.922, p < 0.001, η^2^ = 0.756, Fig. 4E) were lower while the level of HDL (F_(1,28)_ = 87.585, p < 0.001, η^2^ = 0.758, Fig. 4D) was higher in HIIT group. Pre- and post-intervention, the within-group comparisons showed that HIIT group had markedly decreased TC (p < 0.001), TG (p < 0.001), LDL (p < 0.001), and TC/HDL (p < 0.001), but had markedly increased HDL (p < 0.001). However, Control group had obviously increased TG (p = 0.012) and LDL (p = 0.032), no significant difference in TC, HDL, and TC/HDL (Fig. 4A–E).

### Effects of HIIT on AVI and API

**Fig. 5:**
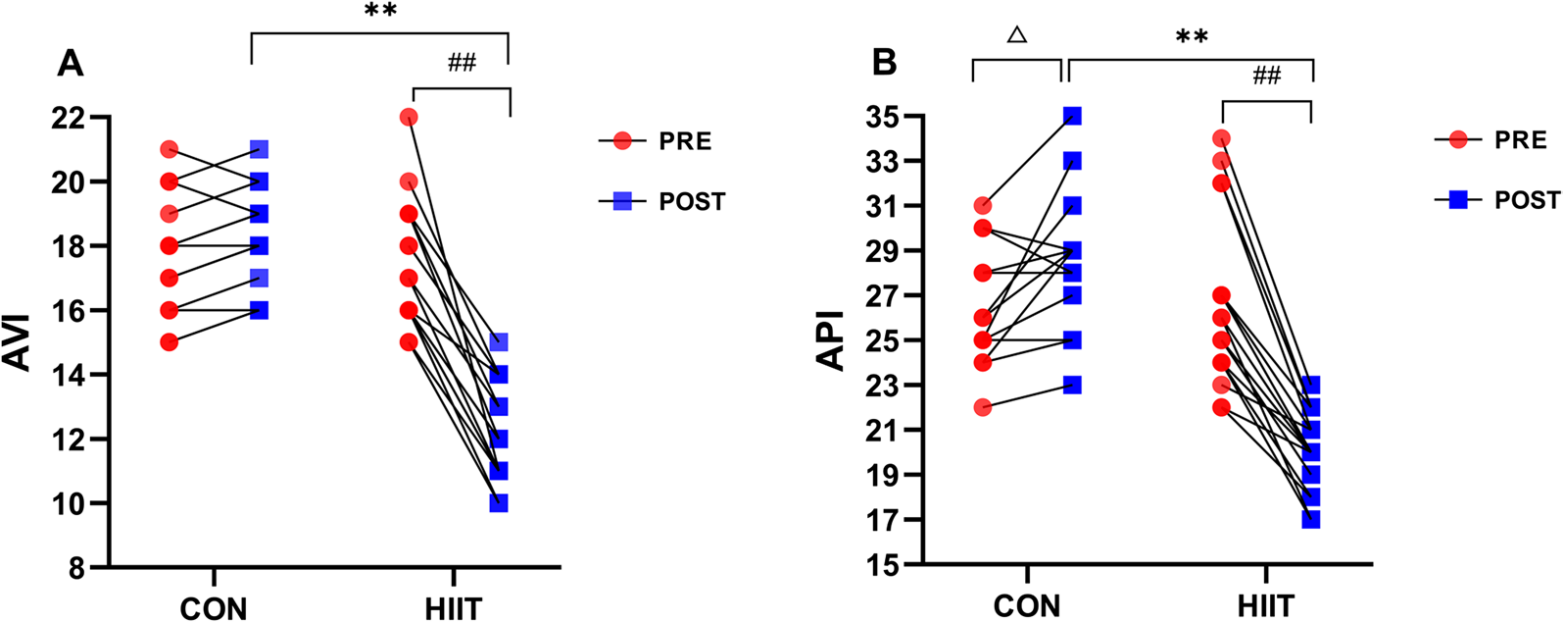
Effects of HIIT on AVI and API. Changes of **A** AVI (arterial velocity pulse index) and **B** API (arterial pressure volume index) were examined pre- and post-experiment using PASESA AVE-2000 PLUS. n = 13 Control group, n = 17 HIIT group. Repeated measures ANOVA followed by simple effect test analysis were used. Data are presented as mean ± SE. ^*^p < 0.05, ^**^p < 0.01, HIIT group (post) vs. Control group (post); ^##^p < 0.01, pre vs. post in HIIT group; ^△^p < 0.05, pre vs. post in Control group

We found a significant time × group interaction in the aspects of AVI (F_(1,28)_ = 98.496, p < 0.001, η^2^ = 0.779) and API (F_(1,28)_ = 66.458, p < 0.001, η^2^ = 0.704). There was no significant difference in HR, SBP, and DBP between the two groups pre-HIIT intervention. However, simple effect test analysis found that compared with the control subjects post-HIIT intervention, both AVI (F_(1,28)_ = 103.892, p < 0.001, η^2^ = 0.788, Fig. 5A) and API (F_(1,28)_ = 83.242, p < 0.001, η^2^ = 0.748, Fig. 5B) were obviously decreased in HIIT group. Pre- and post-intervention, the within-group comparisons showed that HIIT group had obviously decreased AVI (p < 0.001) and API (p < 0.001), while Control group only had significantly increased API (p = 0.015) but no difference in AVI (Fig. 5A–B).

## Discussion

There is strong evidence linking NWO and increased CVD and all-cause mortality, and women with NWO are 2.2 times more likely to die from CVD than those with low BF% [1]. Female university students have a high incidence of NWO but are underdiagnosed and understudied. In this study, we used the bioelectrical impedance analyzer InBody-770 to measure and estimate body composition and found some significantly progressive adverse changes in female university students with NWO, such as increased body weight, BMI, BF%, BFM, BFM of the left arm, and obesity degree, which may lead to potential cardiovascular health hazards. Numerous early numerous studies validated that elevated BF% and BFM are associated with disturbances in lipid metabolism and cardiometabolism [28], which can impair cardiovascular function. Herein, we also found that the control group had obviously increased resting HR, SBP, DBP, TG, and LDL levels over time. The normal ranges of SBP and DBP were less than 120 and 80, respectively; however, both SBP and DBP were excessive in females with NWO, implying risk of hypertension. One study pointed out that subjects with NWO demonstrated left ventricle systolic and diastolic dysfunction, and increased fibrosis intensity [29], so we recommend that both systolic and diastolic BP should be evaluated to better define the metabolic and vascular profile of females with NWO.

Previous evidence has shown that higher BMI, BP, TG levels, and LDL levels may cause persistent endothelial damage and lead to endothelial dysfunction and increases in AS, which are considered the first steps in the progression of atherosclerosis [30]. An elevated HR is an important determinant of the mechanical properties of vascular atherosclerotic lesions, which are related to the development and progression of vasculopathy [31, 32]. Two key points of HR elevation contribute to atherosclerosis. First, an elevated HR increases oscillatory shear stress to lower the arterial distensibility. Second, it intensifies the pulsatile motion of the heart to hinder local hemodynamics [33]. BP has been reported to be mediated by AS [34]. For example, an elevated SBP is significantly associated with higher central arterial stiffness in normotensive men and women [35].

Blood-based biomarkers such as TC, TG, LDL, and HDL levels are well-established indicators of cardiovascular function risk. In this study, we found that the values of TC (suitable range < 5.20 mmol/L, edge elevated 5.20–6.20 mmol/L), TG (suitable range < 1.70 mmol/L, edge elevated 1.70–2.30 mmol/L), and LDL (suitable range 2.60–3.40 mmol/L, edge elevated 3.40–4.10 mmol/L) were within the edge elevated range, and HDL (suitable range ≥ 1.00 mmol/L, abnormal range < 1.00 mmol/L) was within the abnormal range in the two groups before the experiment. Similarly, these indicators were further elevated or abnormal in the control group over time. Atherosclerosis is characterized by visible atherosclerotic lesions in the artery walls, especially in the coronary artery and aorta. These lesions primarily occur due to excessive lipid deposition [36]. Therefore, atherosclerosis originates from the passive diffusion of circulating LDL through endothelial junctions into the vessel intima [37]. Two of the most representative indicators are elevated levels of TC and LDL [36]. The reduction in LDL can further lower cardiovascular mortality. TG is the major component of triglyceride-rich lipoproteins (TRLs), which needs to combine with associated proteins into lipoprotein particles [38]. Recently, some studies have reported that TG and TC contents within TRLs may contribute to the development of atherosclerotic cardiovascular disease [39, 40]. In addition, HDL-enhancing foam cell cholesterol efflux is considered the first step of reversing TC transport, which is a promising antiatherogenic strategy [41]. The sensitivity of the TC/HDL value is higher than that of pure TC or HDL values, and is used to independently predict obesity, atherosclerosis, and coronary heart disease [42].

AS is considered one of the earliest detectable measures of vascular damage. However, it is very difficult to directly measure AS. Recently, the AVI and API, two novel noninvasive vascular indices for evaluating systemic and peripheral AS, respectively, have been gaining attention. They are examined using cuff oscillometric technologies and suprasystolic cuff oscillometric waves in the clinical setting. The AVI is significantly associated with BMI, abdominal circumference, and TG levels [43]. The API is affected by coronary stenosis and arterial compliance and is more sensitive to arterial SBP [44].

The AVI and API have emerged as surrogate markers of cardiovascular disease in obesity and as predictors for CVD events in adulthood. According to the manufacturer’s instructions, the normal range of the AVI is 11 to 15 (age 18–23 years), and the API is 15 to 22 (age 15–22 years). In the current study, we found that both the AVI and API exceeded the normal range in the two groups. Even worse, the API was further increased in the control group because they did not receive effective intervention. Combined with abnormal body composition, BP, and lipid metabolism, these results strongly suggested that the females with NWO have an increased risk of arteriosclerosis and CVD. Early detection of the degree of arteriosclerosis and timely intervention are of great significance for preventing and treating cardiovascular and cerebrovascular diseases in the population with NWO. A previous study revealed that an enhanced AVI represents an increased workload on the heart with elevated central BP and is highly correlated with the augmentation index. The API is a useful predictor of future cardiovascular disease, which is independently associated with both the Framingham Cardiovascular Risk Score and the Suita Score [45]. Fujiwara et al. found that the AVI and API were higher in patients with coronary artery disease and positively correlated with the severity of coronary artery stenosis [13]. Therefore, higher AVI and API values represent a higher propensity for AS in the population with NWO. The potential mechanisms by which NWO increases AS and impairs vascular function may be ascribed to chronic high fat accumulation, which stimulates multiple proinflammatory cytokines, elevates NOX-mediated ROS production, induces oxidative stress, provokes vascular endothelial cell functional disorder, and disturbs the secretion of vascularizing factors (nitric oxide synthase and nitric oxide) and vasoconstrictor factor (Ang-II and endothelin, vasoconstrictor) [46, 47].

Prescribing physical activity to people with obesity is no longer a rarity and in fact has become recognized as a necessity. In recent years, HIIT has been among the most highly recommended measures for obesity because it is well tolerated and favorably affects cardiometabolic risk factors. Our study showed that 4-week HIIT effectively reduced the body weight, BMI, BF%, BFM, LBFM, and obesity degree, and increased SMM, protein content, BMC, FFM, LFFM, TBW, LTBW, BCM, and the InBody score; only LMC had no difference. Interestingly, after the HIIT intervention, most of the results of the between-group comparisons were in line with the within-group comparisons of the HIIT group except there were differences in body weight, BMC, LFFM, or LTBW. The main reason may be due to the difference in sample size between the two groups (HIIT, n = 17 *vs.* Control, n = 13). It is also possible that the statistical method of repeated-measures ANOVA may be overly strict. Nevertheless, based on these results, we were still able to demonstrate that short-term HIIT could be a potent stimulus for effectively improving body composition in females with NWO. Previous evidence allows us to speculate that these positive improvements in body composition after HIIT were likely the result of an upregulation of bioenergetic oxidation (especially fat oxidation) and energy expenditure due to excess postexercise oxygen consumption (EPOC) [48, 49]. HIIT leads to the consumption of large amounts of glycogen during exercise, so more fat is oxidized during the recovery period to resynthesize glycogen. HIIT training may mobilize some skeletal muscles, which may also activate skeletal muscle catabolism signaling mechanisms (especially lipodieresis) and increase muscle protein synthesis [50, 51]. Besides these, HIIT could promote the secretion of catecholamines, epinephrine, norepinephrine, and growth hormones, which can accelerate fat decomposition to achieve effective fat and body weight loss [25].

Previous studies elucidated that HIIT could decrease BMI, BF, and BFM in overweight and obese individuals [21, 52], and HIIT elicited superior benefits than MICT in weight control, fat loss (especially abdominal and visceral fat), FFM, and SMM in both healthy and chronically ill populations [53–55]. HIIT has even been shown to reduce epicardial adipose tissue mass and cardiovascular risk in physically inactive participants with abdominal obesity [56]. Even 14-week home-based HIIT can produce improvements in risk factors for cardiovascular and metabolic diseases in overweight/obese women [57]. The results imply that HIIT is a sustainable training strategy for improving weight management. Furthermore, although generating a similar magnitude of modest improvements in BFM and waist circumference as MICT does in overweight and obese individuals, HIIT can save nearly 40% time commitment each week [58]. Therefore, HIIT is considered a time-efficient exercise strategy for managing overweight and obesity.

However, to the best of our knowledge, little is known regarding the effects of HIIT on CVD factors, namely, BP, lipid metabolism, and AVI/API, in female university students with NWO. First, BP is closely correlated with cardiovascular health, and hypertension has led to high cardiovascular morbidity and mortality worldwide [59]. Some studies have shown that HIIT had a superior function of decreasing resting HR, SBP, and DBP [60–62] and was associated with greater improvements in dealing with hypertension when compared to MICT among hypertensive patients and overweight/obese adolescent girls [63–65]. These results are concordant with our findings. Second, HIIT has been reported to have the function of reducing lipid metabolism disorders in obesity [66, 67] and may be a preferable therapy for atherosclerosis. Our results demonstrated that 4-week HIIT could significantly reduce TC, TG, LDL, and TC/HDL levels and raise HDL levels in the HIIT group. More importantly, HIIT could adjust these indexes to the normal ranges, and our finding was consistent with that of Fisher et al. in overweight and obese young men [68]. Gripp et al. reported that most of the positive effects of HIIT were also found to be longer-lasting and maintained after suspension for 4 weeks [69]. The accumulation of LDL inside the blood vessels serves as a major cause of arteriosclerosis, and HIIT can prevent LDL accumulation [25]. Moreover, HIIT can also affect HDL function, including the promotion of reverse cholesterol transport and lipid peroxide transport clearing [70]. Therefore, a pattern of short-term HIIT will benefit the females with NWO in ameliorating lipid metabolism.

Third, although the effects of aerobic exercise on reducing AS and CVD risk have been investigated before, the relationship between AVI/API values and HIIT improvements in AS in females with NWO has not been fully studied. Here, we examined the effect of HIIT on the two new arterial indices for AS in Chinese female university students with NWO for the first time. We verified that HIIT could significantly lower values of the AVI and API to the normal ranges. AS is mainly influenced by vascular endothelial function, and HIIT has well-established beneficial effects on endothelial function in overweight men/adolescents and obese young women [71, 72]. HIIT has been reported to increase endothelial eNOS protein content and NO availability and cause significant improvements in brachial artery endothelial-dependent dilatation and aortic stiffness in obese individuals with elevated CVD risk [73]. Additionally, a meta-analysis showed that HIIT was associated with up to a twofold increase in endothelial dilator function at the macrocirculatory level when compared with MICT in adults with metabolic and cardiovascular disease [74].

However, the mechanism of reducing the propensity for AS is still unclear in our study. There are many gaps in the literature on NWO, unlike overt obesity, and further study should disclose the underlying relationship between HIIT and vascular adaptation in improving AS in female university students with NWO and explore the molecular mechanisms. It should be noted that the exercise risk assessment needs to be carried out before the formulation of an HIIT prescription, and adaptive training along the increasing intensity and sufficient warm-up, relaxation, and cool downs are essential for avoiding sports fatigue and sports injuries and even the concurrent health risk. In this study, we did not observe any adverse exercise events (e.g., muscle injury, overfatigue, syncope, palpitation, angina, abnormal fluctuation of blood pressure, nausea, vomiting, dyspnea) during the HIIT intervention. Hence, HIIT utilization is safe for females with NWO. Last but not least, large, multicenter, and prospective studies are required to establish the optimal HIIT protocols for female university students with NWO.

## Limitations

The study was designed as a prospective, randomized controlled trial (RCT) exercise intervention trial comparing the effects of 4-week HIIT program on female university students with NWO. However, this study has some limitations that should be considered before interpreting the results. First, the sample size was relatively small and the control group had a higher attrition rate (35%), probably because they didn’t see the weight loss or benefit. In contrast, the HIIT group had high adherence. Even so, it did not prevent us from seeing clear differences between the two groups, and the magnitude of the beneficial effects of HIIT on NWO female students’ AS was adequate. Second, although it has been reported that HIIT may result in suppressed appetite [75], participants were instructed to not alter their diet behaviors for the duration of the intervention, change in habitual energy intake was not monitored, which may affect the final results. Third, we did not examine the arterial compliance, reflected wave, vascular endothelial function, or female hormones, which may have important effects on AS. Forth, we did not use DXA to diagnose obesity and body composition due to it is quite expensive, time-consuming, and requires specialized radiology equipment and environment, although it is the gold standard. We used InBody 770 to take place of DXA in this study, which also has high accuracy. Fifth, the blood samples were not taken and analysed in a laboratory, although this method has high accuracy. We used three-in-one Cholesterol Monitoring system CCM-111 to take place of laboratory testing due to it only requires a little fingertip blood sample (35 μL) and has rapid test speed (≤ 2 min) and a relatively high accuracy.

## Conclusion

All in all, our observations suggest that HIIT is an effective and acceptable activity for female university students with NWO, and we provide up-to-date evidence on the impact of short-term HIIT in obviously improving body composition and abnormal lipid metabolism, reducing BP and AVI/API, as expected. These changes caused by HIIT will be hugely beneficial for reducing AS and CVD risk and enhancing the physical fitness and well-being of female university students with NWO.

## Data Availability

The datasets used and/or analyzed during the current study are available from the corresponding author on reasonable request.

## Abbreviations

HIIT: High intensity interval training
NWO: Normal weight obesity
BMI: Body mass index
AS: Arterial stiffness
HR: Heart rate
BP: Blood pressure
SBP: Systolic blood pressure
DBP: Diastolic blood pressure
AVI: Arterial velocity pulse index
API: Arterial pressure volume index
CVD: Cardiovascular disease
BF%: Body fat percentage
VAT: Visceral adipose tissue
TC: Total cholesterol
TG: Triglyceride
LDL: Low-density lipoprotein-cholesterol
HDL: High-density lipoprotein-cholesterol
CAVI: Cardio Ankle Vascular Index
baPWV: Brachialankle Pulse Wave Velocity
IMT: Intima-media thickness
aAI: Aortic Augmentation Index
MICT: Moderate-intensity continuous training
BFM: Body fat mass
LBFM: BFM of left arm
SMM: Skeletal muscle mass
BMC: Bone mineral content
FFM: Fat free mass
LFFM: FFM of left arm
TBW: Total body water
LTBW: Total body water of left arm
LMC: Measured circumference of left arm
BCM: Body cell mas
TRLs: Triglyceride-rich lipoproteins
RCT: Randomized controlled trial
